# Choosing statins: a review to guide clinical practice

**DOI:** 10.20945/2359-3997000000306

**Published:** 2020-11-09

**Authors:** Roberta de Pádua Borges, Nathália Abi Habib Degobi, Marcello Casaccia Bertoluci

**Affiliations:** 1 Universidade Federal do Rio Grande do Sul Porto Alegre RS Brasil Programa de Pós-Graduação em Endocrinologia, Universidade Federal do Rio Grande do Sul (UFRGS), Porto Alegre, RS, Brasil; 2 Universidade Federal do Rio Grande do Sul Porto Alegre RS Brasil Programa de Pós-Graduação em Ciências Médicas, Universidade Federal do Rio Grande do Sul (UFRGS), Porto Alegre, RS, Brasil; 3 Universidade Federal do Rio Grande do Sul Hospital de Clínicas de Porto Alegre Porto Alegre RS Brasil Serviço de Endocrinologia, Hospital de Clínicas de Porto Alegre (HCPA), Universidade Federal do Rio Grande do Sul (UFRGS), Porto Alegre, RS, Brasil; 4 Universidade Federal do Rio Grande do Sul Faculdade de Medicina Departamento de Medicina Interna Porto Alegre RS Brasil Departamento de Medicina Interna, Faculdade de Medicina, Universidade Federal do Rio Grande do Sul (UFRGS), Porto Alegre, RS, Brasil

**Keywords:** Statins, cardiovascular disease, cholesterol, type 2 diabetes

## Abstract

Statins are among the most widely prescribed medicines in the world and have proved their value in reducing cardiovascular events and mortality. Many patients report adverse effects that lead to interruption of treatment. This review aims to individualize statin treatment, considering efficacy for reducing cardiovascular risk and safety, in the setting of specific diseases, to minimize the side effects and improve compliance. We gathered evidence that may help clinicians to choose specific statins in different clinical situations, such as the risk of new diabetes, chronic kidney disease, liver disease, human immunodeficiency virus infection, organ transplant, heart failure and elderly people. Efficacy of statins is well established in a large number of clinical conditions. Therefore, main objective is to revise statin in specific clinical settings, based on pharmacokinetics, safety, drug metabolism and interactions to provide the best choice in different clinical scenarios.

## INTRODUCTION

According to the Centers for Disease Control and Prevention ( 1 ), more than 25% of adults above 40 years of age, living in United States, use statins. The efficacy and effectiveness of statins are unquestionable. They reduce mortality and cardiovascular events in patients at low, intermediate, and high cardiovascular risk ( 2 , 3 ). However, numerous users report muscle symptoms that lead to interruption of treatment. This is perceived as a barrier in maintaining long-term adherence to statins ( 4 ). Although the risk of myopathy reported in randomized clinical trials is around 1-4 per 10,000 individuals over five years ( 3 ), the incidence of statin-associated muscle symptoms in real-life is much greater, reaching 10%-20% ( 4 ). This discrepancy may result from selection bias in clinical trials where the best adherent patients are preferably included in the study after the run-in phase.

Changing the statin type and dosage after a brief washout period is usually recommended to mitigate statin intolerance ( 4 ). However, choosing statins wisely may be a better strategy to reduce side-effects and to improve adherence. To achieve these, this review aims to rationalize statin treatment, considering the cardiovascular risk profile, the presence of significant comorbidities, as well as the drug to drug interactions.

## Statins and new onset diabetes

### Can statins cause diabetes?

Development of type 2 diabetes mellitus (T2DM) in patients taking statins is well defined in many clinical trials. In the Justification for the Use of Statins in Prevention: an Intervention Trial Evaluating Rosuvastatin (JUPITER) trial ( 5 ), 17,802 individuals without a history of diabetes or cardiovascular disease were assigned to rosuvastatin 20 mg or placebo and followed up for a median of 1.9 years. The incident of T2DM was 25% higher in the rosuvastatin group (hazard ratio (HR) 1.25, 95% confidence interval (CI) 1.05-1.49, *p* = 0.01). The risk for T2DM was associated with advanced age, increased fasting blood glucose, and metabolic syndrome. More recently, a meta-analysis ( 6 ) included 14 randomized clinical trials with a total of 94,943 participants. Of these, 4,599 developed incident diabetes during a 4-year follow-up. The overall odds-ratio (OR) was higher in statin users compared with the controls (OR 1.11; 95% CI 1.0-1.2; *p* = 0.007). Thus, it is estimated that around 10%-11% of patients would develop T2DM by using statins.

### How statins cause diabetes?

Statins can cause T2DM via multiple possible mechanisms. They may: 1) impair insulin secretion by changes in the calcium channel system in pancreatic beta-cells (7); 2) reduce the translocation of glucose transporter type 4 (GLUT-4) in target cells (8); or 3) decrease the downstream products of cholesterol (such as coenzyme Q10, farnesyl phosphate, geranylgeranyl pyrophosphate and dolichol, consequently decreasing intra-cellular signaling ( 9 , 10 ). Studies have also proposed decreases in adiponectin and leptin levels and adipocyte differentiation ( 11 , 12 ).

### Which statins have greater risk for developing new T2DM?

**Figure 1:**
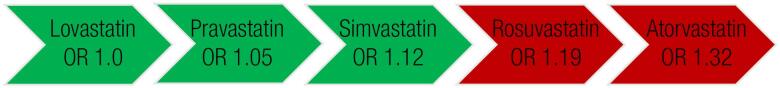
Progressive risk of development of diabetes with statin use

Generally, the more potent the statin, the greater the risk for developing T2DM. In a sub-group of a meta-analysis ( 6 ), the most potent statins, atorvastatin and rosuvastatin, were most firmly associated with increases in risk for T2DM (OR 1.29; *p* = 0.042 and OR: 1.17; *p* = 0.01, respectively. Figure 1 displays the ranked OR of statins for causing new diabetes obtained from this meta-analysis ( 6 ). Additionally, low-potency statins, such as pravastatin and pitavastatin, may have a much less impact in glucose metabolism. Another meta-analysis ( 13 ) revealed that pravastatin significantly improved insulin sensitivity. Pitavastatin, when compared with placebo, did not affect hepatic or whole-body insulin sensitivity ( 14 ).

The risk of new-onset diabetes, however, must be weighed against the risk of cardiovascular events and mortality. In a pooled analysis ( 15 ) including 5 large trials with 32,752 participants, intensive statin therapy was associated with an increased risk to incident diabetes compared with moderate statin therapy (OR: 1.12; 95% CI 1.04-1.22). However, the number needed to harm for intensive-dose statin therapy to cause new-onset diabetes was 498 per year, while the number needed to treat (NNT) to prevent cardiovascular events with the same statin therapy was 155. In conclusion, one should not avoid high-potency statins for the fear of causing diabetes in patients at high and very-high risks.

### Can statins affect glucose control in patients with diabetes?

A meta-analysis of randomized clinical trials ( 16 ) with 6,875 participants compared statin therapy with placebo in T2DM. It found that the treatment with atorvastatin may worsen glycated hemoglobin (HbA1c) levels (Standard Mean Deviation (SMD) 0.12%, 95% CI 0.06-0.18; *p* = 0.000). However, there was no difference with rosuvastatin, lovastatin, pravastatin, and fluvastatin treatments compared with placebo.

A network meta-analysis ( 17 ) with 23 trials with 2,703 patients provides the best evidence by comparing the effects of different statin strategies on glycemic control in patients with T2DM. HbA1c and fasting plasma glucose were determined. Overall, statins were associated with an increased level of HbA1c than the placebo. However, high-intensity atorvastatin significantly worsened the glycemic control outcomes, while moderate-intensity pitavastatin significantly reduced the HbA1c and fasting blood glucose levels in patients with T2DM. Pitavastatin might be a better choice, if considering only the control of diabetes. It is crucial, however, to consider the cardiovascular risk of the patient. The greater the cardiovascular risk, more important it is to use high-intensity statins, despite the potential worsening of glycemic control.

## Statins and human immunodeficiency virus (HIV)

### Are HIV infected people at increased cardiovascular risk?

People living with HIV have an increased risk for myocardial infarction, independent of the traditional cardiovascular risk factors. In this population, dyslipidemia is observed in up to 80% of the individuals ( 18 ). Mechanisms for increased cardiovascular risk in HIV are multifactorial, including prolonged immunosuppression, chronic inflammation, anti-retroviral therapy (ART), and dyslipidemia ( 19 ). The use of ART in HIV patients is especially important when considering the use of statins. In a meta-analysis ( 20 ), the relative risk (RR) of cardiovascular events among patients without ART was 1.61 (95% CI 1.43-1.81; *p* < 0.001) while that in patients with the treatment was 2.0 (95% CI 1.7-2.37; *p* < 0.001), both compared with HIV-uninfected people. The RR of myocardial infarction for protease inhibitors (PI) treatment vs. non-PI treatment was 1.41 (95% CI 1.2-1.65) ( 18 , 21 , 22 ).

### Do statins reduce cardiovascular risk in people with HIV?

Statins reduce mortality in people living with HIV. In a meta-analysis ( 23 ) of observational studies including 35,708 patients, statin use was associated with a 33% reduction in all-cause mortality. The HIV-Infected Patients and Treatment with Pitavastatin vs Pravastatin for Dyslipidemia (INTREPID) study ( 24 ) was a randomized, double-blind, superiority trial that compared pitavastatin with pravastatin in HIV patients on ART for at least a six-months and presenting dyslipidemia. Low-density lipoprotein cholesterol (LDLc) reduction was 31.1% with pitavastatin and 20.9% with pravastatin (p < 0.0001) at 12 weeks. No serious adverse event was considered treatment related.

### Which statins are safe to use along with ART?

Cytochrome P450 via CYP3A4 metabolizes most of the statins; many antiretroviral drugs are also eliminated by this same pathway. Therefore, this could result in potential harm due to drug interactions with greater adverse risk for rhabdomyolysis and renal failure ( 25 ). For this reason, individuals receiving treatment with PI should avoid using simvastatin and lovastatin. On the other hand, atorvastatin, rosuvastatin, fluvastatin, and pravastatin can be recommended with dose adjustments, according to the type of ART ( 26 ) ( Table 1 ).

**Table 1:** Interactions between statins and HIV anti-retroviral therapy: protease inhibitors (PI), non-nucleoside reverse transcriptase inhibitors (NNRTI), and integrase strand transfer inhibitors (ISTI) (26)

Statin	PI						NNRTI				ISTI
	ATV	ATV/c	DRV/r	DRV/c	LPV/r	TPV/r	EFV	ETR	NVP	RPV	EVG
Atorvastatin	10		20	20	20		*	*	*		20
Lovastatin							*	*	*		
Pitavastatin											
Pravastatin							*				
Rosuvastatin	10	10		20	10						
Simvastatin							*	*	*		
Fluvastatin											

The number inside the cell indicates the maximum statin dose (mg) recommended while in the presence of the corresponding drug.

* May require higher starting dose but should not exceed the maximum recommended dose. ATV: atazanavir; c: cobicistat; DRV: darunavir; r: ritonavir; LPV: lopinavir; TPV: tipranavir; EFV: efavirenz; ETR: etravirine; NVP: nevirapine; RPV: rilpivirine. EVG: elvitegravir.

**Table:** 

	No dose-adjustment necessary.
	Titrate dose carefully and use the lowest necessary dose.
	Co-administration not recommended.

Most non-nucleoside reverse transcriptase inhibitors may induce the metabolism of atorvastatin via CYP3A4, decreasing overall lipid reduction; therefore, higher doses of statins may be needed. Rosuvastatin can interact with PI through non-CYP mechanisms ( 27 ). Pitavastatin is the only statin without significant drug interactions and the need for dose adjustments. No dose modifications are necessary for the nucleoside reverse transcriptase inhibitors and for the CCR5 receptor inhibitor ( 27 ).

## Statins and chronic kidney disease (CKD)

### Is cardiovascular mortality increased in patients with CKD?

Patients with CKD are at an increased risk for cardiovascular mortality, which is proportional to the fall in estimated glomerular filtration rate (eGFR) and the increase in albuminuria. A meta-analysis including 105,872 participants in 14 studies revealed that the HR for all-cause mortality and cardiovascular mortality was constant when eGFR and albumin-to-creatinine ratio were in the normal range. However, it increased progressively when eGFR was progressively lower. When compared with patients with normal eGFR, the HR for all-cause mortality in patients at eGFR 60, 45 and 15 mL/min/1.73m^2^ were 1.18 (95%CI 1.05-1.32); 1.57 (95%CI 1.39-1.78) and 3.14 (95%CI 2.39-4.13), respectively. Results were similar for cardiovascular mortality ( 28 ). When starting dialysis, however, cardiovascular and non-cardiovascular mortality rates are much increased, being 8.8 (95%CI, 8.6-9.0) and 8.1 (95%CI, 7.9-8.3) times higher than in the general population, respectively ( 29 ).

Dyslipidemia is also a major risk factor for cardiovascular disease in patients with CKD ( 30 ). Several mechanisms increase cardiovascular risk, such as activation of inflammatory cell-signaling pathways, intrarenal atherogenesis, and cellular impairment in the microvasculature ( 31 ).

### Do statins reduce cardiovascular events and mortality in patients with CKD not receiving dialysis?

In patients with CKD not receiving dialysis, the reductions in major cardiovascular events due to statins is proportional to reductions in LDLc levels. The Study of Heart and Renal Protection (SHARP) trial ( 32 ) was a randomized double-blind trial, including 9,270 patients with moderate to severe CKD without previous myocardial infarction or coronary revascularization. Patients were assigned to either simvastatin 20 mg with ezetimibe or placebo. After a median follow-up of 4.9 years, the treatment group achieved a 33 mg/dL lowering in their LDLc levels, resulting in a 17% proportional reduction in major atherosclerotic events. There was also a 25% reduction in non-hemorrhagic strokes (RR 0.75, 95% CI 0.60-0.94, *p* = 0.01).

A meta-analysis of individual data ( 30 ) with 183,419 patients from 28 trials assessed the effects of statins on major vascular events, (non-fatal myocardial infarction, coronary death, stroke, or coronary revascularization) in patients stratified by eGFR. Statin treatment reduced the risk of first major vascular event in 21% per mmol/L of reduction in LDLc ( *p* < 0.0001). The benefit decreased as eGFR declined, especially in patients with eGFR < 30 mL/min/1.73 m^2^ and patients on dialysis. This effect was also observed for major coronary events ( *p* = 0.01) and vascular mortality ( *p* = 0.03).

Another meta-analysis ( 33 ) assessed major cardiovascular events, all-cause mortality and cardiovascular mortality. This included studies that compared two different statin regimens in patients with CKD not receiving dialysis, and studies with statins against placebo. In this analysis, statin therapy consistently prevented major cardiovascular events (RR 0.72, 95% CI 0.66-0.79), all-cause mortality (RR 0.79, 95% CI 0.69-0.91) and cardiovascular mortality (RR 0.77, 95% CI 0.69-0.87) and myocardial infarction (MI) (RR 0.55, 95% CI 0.42-0.72).

### Which statins reduce proteinuria?

Some but not all statins reduce proteinuria. The Prospective Evaluation of Proteinuria and Renal Function in Diabetic Patients with Progressive Renal Disease (PLANET I) study ( 34 ) was a randomized, double-blind, parallel-group trial that assessed the renal effects of atorvastatin 80 mg and rosuvastatin 10 or 40 mg in patients with diabetes and proteinuria. In the atorvastatin group, proteinuria decreased by 18% (p = 0.003) in 52 weeks from baseline, but there was no fall in the rosuvastatin group, despite a 54% reduction in LDLc. In the post-hoc analysis, the eGFR remained stable with atorvastatin, but significantly decreased in the rosuvastatin group (p = 0.036). Additionally, a meta-analysis of RCTs ( 35 ) in patients with CKD demonstrated that pitavastatin and pravastatin may reduce albuminuria compared with placebo.

### Can statins delay the loss of renal function?

The effects of statins in preserving renal function are not yet well understood and seem to differ according to the type of statin used. In the PLANET 1 study ( 34 ), patients aged 18 years old, with type 1 or type 2 diabetes with proteinuria took angiotensin converting enzyme inhibitors, angiotensin II receptor blockers, or both. Participants were assigned to atorvastatin 80 mg, rosuvastatin 10 mg, or rosuvastatin 40 mg for 52 weeks. The primary endpoint was the change in mean urine protein/creatinine ratio from baseline to week 52 in each treatment group, and eGFR was the secondary outcome. The study enrolled 353 patients. Mean eGFR at 52 weeks was similar to baseline in the atorvastatin 80 mg group at around 68-73 mL/min/1,73 m^2^. During follow-up eGFR decreased significantly in the rosuvastatin 10 mg and in rosuvastatin 40 mg groups whereas it was well-preserved in the atorvastatin group. Thus, atorvastatin but not rosuvastatin may have reno-protective effect in CKD population ( 34 ).

On the other hand, the SHARP study did not report a synergistic effect of the association of simvastatin and ezetimibe on preventing renal function loss. In a prespecified sub-analysis ( 36 ), the main renal outcome was end-stage renal disease, defined as the initiation of dialysis or renal transplantation; this analysis shows that daily use of simvastatin 20 mg plus ezetimibe 10 mg was beneficial. The lowering of LDLc by 38 mg/dL within five years, in 6245 individuals with CKD not receiving dialysis, did not slow the progression of kidney disease ( 36 ).

### Are statins efficacious in preventing cardiovascular disease in patients receiving dialysis?

The efficacy of statins in the prevention cardiovascular events in patients receiving dialysis is controversial. The Die Deutsche Diabetes Dialyse Studie (4D) ( 37 ), is a placebo-controlled trial including 1,255 patients with T2DM who received maintenance hemodialysis. In this, atorvastatin 20 mg lowered the LDLc levels by 42%, but there was no significant reduction of the primary composite endpoint- cardiac death, myocardial infarction and stroke. Curiously, fatal strokes increased in the atorvastatin group (RR, 2.03; 95% CI 1.05-3.93; *p* = 0.04).

Another randomized clinical trial: A Study to Evaluate the Use of Rosuvastatin in Subjects on Regular Hemodialysis: An Assessment of Survival and Cardiovascular Events (AURORA) trial ( 38 ), enrolled individuals of age 50-80 years, receiving dialysis for at least three months and administered them with either placebo or rosuvastatin (10 mg). The primary outcome was the time to major adverse cardiovascular events (MACE). At the end of the study, 396 patients in the treatment group and 408 patients in the placebo group reached the primary end point (9.2 and 9.5 events per 100 patient-years, respectively; HR, 0.96; 95% CI 0.84-1.11; *p* = 0.59). These two studies raise a strong evidence suggesting patients already on dialysis do not benefit from initiating statin treatment.

However, Brazilian guidelines on prevention of cardiovascular disease in patients with diabetes ( 39 ) recommend not to suspend statin treatments when starting dialysis in the patients already using statins. In a retrospective cohort study ( 40 ) with 14,298 participants, patients who were on statins and continued using it for at least six months during the first year of dialysis had a 28% lowered risk of death (HR, 0.72; 95% CI 0.66-0.79) and a 18% lower risk of fatal cardiovascular events (adjusted HR, 0.82; 95% CI 0.69-0.90) during the subsequent 12 months compared with patients who discontinued the use.

### Are statins safe in renal failure?

Most statins depend on renal excretion. At some level, an impaired renal function could lead to increased systemic exposure with potential toxicity. However, in general, renal excretion is low, with the exception for pravastatin ( 41 ). Statins are well tolerated in CKD, and dose adjustments according to renal function ( 42 ) should be followed to avoid adverse events ( Table 2 ).

**Table 2:** Maximum recommended doses of statins in adults with chronic kidney disease according to estimated glomerular filtration rate (eGFR)

Statin	eGFR mL/min/1.73 m^2^				
	60-89	45-59	30-44	15-29	<15
Atorvastatin					
Lovastatin				20	20
Pitavastatin		2	2	2	2
Pravastatin		10	10	10	10
Rosuvastatin				10	10
Simvastatin				10	10
Fluvastatin					
Simvastatin/ezetimibe		20/10	20/10	20/10	20 /10

**Table:** 

	No dose adjustment necessary.
	Maximum dose (mg).

## Statins in recipients of organ transplantation

### Kidney transplant

Cardiovascular disease is a leading cause of death in renal transplant recipients (RTR) ( 43 ). This is attributable to a combination of conventional cardiovascular risk factors, such as hypertension, diabetes, dyslipidemia, and the metabolic effects of immunosuppressant treatments ( 44 ). Immunosuppressants include antiproliferative agents, calcineurin inhibitors, mammalian target of rapamycin (mTOR) inhibitors, and corticosteroids.

The Assessment of Lescol in Renal Transplantation (ALERT) trial ( 45 ) was a large multicenter study that assessed cardiac and renal outcomes in 2,102 RTRs followed for five years. Treatment with fluvastatin (40-80 mg) lowered LDLc by 39 mg/dL (1 mmol/L), with a reduction of 38% in the risk of cardiac death and 32% in the risk of non-fatal myocardial infarction. Fluvastatin was well tolerates, and adverse events were similar among groups, despite high risk of drug interactions in this population, especially considering that they were treated with cyclosporine. A Cochrane meta-analysis ( 46 ), including 22 studies, enrolling 3465 RTRs, comparing statins with placebo, supports their benefits on cardiovascular outcomes. However, the doses used were lower than usual (10 mg simvastatin equivalent).

### Cardiac transplant

A meta-analysis ( 47 ) involving 2,295 patients assessed the benefits of statins on survival in cardiac transplant recipients. There was a reduction in all-cause mortality (OR 0.26; 95% CI 0.20-0.35; *p* < 0.0001) with statin use. Additionally, there was a decrease in the odds of fatal allograft rejection (OR 0.37; 95% CI 0.21-0.65 *p* = 0.0005), incidence of coronary vasculopathy (OR 0.33 95% CI 0.16-0.68, *p* = 0.003), and terminal cancer (OR 0.30; 95% CI 0.15-0.63, *p* = 0.002). Most statins used were in use of low or moderate-intensity statins.

Pravastatin may have specific benefits in cardiac transplantation. A randomized trial ( 48 ) compared the use of pravastatin with placebo early after cardiac transplantation. The results demonstrated less cardiac rejection, ( *p* = 0.005), lower incidence of graft coronary vasculopathy ( *p* = 0.049), and better survival rates (94% *vs.* 78%, *p* = 0.025) in the pravastatin group. In an one-year observational study ( 49 ), pravastatin 40 mg was used in cardiac transplant recipients and was compared with simvastatin 20 mg. Pravastatin was associated with a trend to superior survival at 12 months (97.6 % *vs.* 83.7%, *p* = 0.078) and lower frequency of adverse events. Around 13,3% of the patients developed myositis in the simvastatin group while there was no case in the pravastatin group ( *p* = 0.032). Although these studies should be further confirmed, it seems likely that pravastatin may have specific benefits in cardiac transplantation.

### Liver transplant

Evidence of cardiovascular benefit in liver transplant (LT) due to statins is less clear. A single-center retrospective cohort study ( 50 ) evaluated 495 registries of patients who had undergone LT over a 10-year period. The study addressed if exposition to treatment of dyslipidemia were associated with changes in mortality in this population. They observed patients for a mean follow-up of 4.5 years. There were 96 patients with dyslipidemia before LT and 157 developed dyslipidemia after LT. Statins use was in only 45.7% of patients with coronary artery disease (CAD), and 71.1% were initiated on statins after LT for dyslipidemia. Statin use was independently associated with lower overall mortality (HR 0.25, 95% CI 0.12-0.49; p < 0.001), with no association between previous coronary artery disease or its severity and mortality. Statins were well-tolerated, with only 12% of the patients developing an adverse event requiring the cessation of therapy. Statin type used was not specified in this study.

### Are statins safe with concomitant immunosuppressants drugs?

When using immunosuppressants concomitant with statins, caution is suggested due to possible interactions. Cyclosporine is the one with greatest concern regarding this association. This immunosuppressant is an inhibitor of CYP3A4 at therapeutic doses; therefore, simvastatin, lovastatin, and pitavastatin should be avoided ( 51 ) ( Table 3 ). Further, cyclosporine inhibits statin efflux transport mechanisms, including P-glycoprotein and OATP1B1 ( 51 ). In a double-blind, double-dummy, randomized trial in RTR ( 52 ), there was accumulation of lovastatin but not pravastatin in the blood of cyclosporine-treated patients, since the major metabolites of pravastatin are derived from non-CYP dependent processes. There is a recommendation to implement dosing limits of statins similar to cyclosporine with tacrolimus and mTOR inhibitors because they have similar metabolic pathways ( 53 ) ( Table 3 ).

**Table 3:** Maximum recommended dose of statins when associated with immunosuppressant

Statin (mg)	Cyc	Tac	Siro	Ever	Steroids	MMF	AZA
Atorvastatin		10	10	10			
Lovastatin							
Pitavastatin							
Pravastatin	40	40	40	40			
Rosuvastatin	5	5	5	5			
Simvastatin							
Fluvastatin	40	40	40	40			

Cyc: cyclosporine; Tac: tacrolimus; Siro: sirolimus; Ever: everolimus; MMF: mycophenolate; AZA: azathioprine.

**Table:** 

	No dose-adjustment necessary.
	Maximum dose recommended (mg).
	Co-administration not recommended.

## Statins and heart failure (HF)

### Different phenotypes of HF and statins

Heart failure (HF) usually presents in two main phenotypes: HF with preserved ejection fraction (HFpEF) and HF with reduced ejection fraction (HFrEF). HFpEF is closely related to aging and medical conditions that induce a systemic and microvascular pro inﬂammatory status, such as diabetes, obesity, and hypertension, which are the predominant etiology ( 54 , 55 ). In this phenotype, epicardial adipose volume is increased and is a source of inflammation and damage to the underlying ventricular muscle, impairing its elasticity. This inflammatory process appears to be the primary pathophysiological mechanism of HFpEF, thus, statins may be potentially favorable in these patients, considering their anti-inﬂamatory properties ( 54 , 55 ). In contrast, cardiomyocyte loss and stretch seem to be the principal drivers of HFrEF, while uncontrolled inflammation may not be a major pathophysiological determinant in this condition ( 54 , 55 ). Most guidelines do not advocate initiation of statins in patients with non-ischemic HF; however, maintenance should be considered for those who are already on statins for prevention of coronary artery disease.

### Do statins reduce hard outcomes in patients with reduced ejection fraction?

Two landmark studies CORONA ( 56 ) and GISSI-HF ( 57 ), both large-scale randomized placebo-controlled trials used rosuvastatin in subjects with HF with reduced systolic function, indicated as New York Heart Association (NYHA) class II-IV HF. The CORONA trial ( 57 ) evaluated 5011 individuals, aged ≥ 60 years having HF with reduced ejection fraction of ischemic etiology. Patients received rosuvastatin 10 mg/d or placebo. The primary outcome was a composite of time to death from cardiovascular causes, non-fatal acute myocardial infarctions and non-fatal strokes. They were followed-up for 32.8 months; LDLc decreased by 45%, but there was no difference in the primary outcome or mortality. However in the sub-group analyses of the CORONA trial ( 57 ), rosuvastatin appeared to benefit both the risk of repeated HF admissions and the overall number of admissions ( 57 ).

The GISSI-HF study ( 57 ) enrolled 4574 HF patients of any etiology, aged ≥ 18 years. Rosuvastatin 10 mg/d was tested against placebo for a median follow-up of 46.8 months. The primary endpoint was a composite of time to death and time to death or admission to hospital for cardiovascular reasons. Rosuvastatin again did not affect the clinical outcomes in patients with chronic heart failure of any cause. Thus, there are relatively robust evidences that in patients with HF with reduced ejection fraction, statins may not be useful. However, there are also evidences suggesting that statins could reduce cardiac sympathetic nerve activity and prevent cardiac remodeling in patients with HF ( 58 , 59 ). In a meta-analysis of RCTs in patients only with HFrEF, statins modestly reduced hospitalization for worsening hert failure ( 60 ), although they did not decrease sudden cardiac death ( 61 ). These data, however, still need be confirmed in randomized clinical trials.

### Do statins reduce cardiovascular outcomes in patients with preserved EF (HFpEF)?

The cardiovascular benefits of statins in patients with HFpEF seem to be more promising than in HFrEF. The TOPCAT study ( 62 ) originally was a double-blind, randomized, placebo-controlled trial that included 3,378 individuals with EF>45%, to test the effect of spironolactone against placebo. The primary endpoint was death from cardiovascular causes, aborted cardiac arrest and hospitalization for HF. The mean follow-up was 3.3 years. In a sub-analysis, the authors tried to evaluate the interaction between statin use and history of ischemic heart disease. In this particular type of patient, all-cause death was significantly lower in the group receiving statin therapy compared with the group not taking statins (HR 0.79, 95% CI 0.63-0.99, *p* = 0.04), an effect that was independent of the presence of ischemic heart disease ( 62 ).

Observational data from 9,140 patients with HF and preserved ejection fraction (more than 50%) from the Prospective Swedish Heart Failure Registry was assessed ( 63 ). About 37.5% of the patients were treated with statins, and the interaction between statin use and primary (all-cause mortality) and secondary endpoints were evaluated. Statin therapy was associated with improved outcomes: higher 1-year survival (HR 0.80, 95% CI 0.72-0.89; *p* < 0.001); reduced cardiovascular death (HR 0.86, 95% CI 0.75-0.98; *p* = 0.026), and composite all-cause mortality or cardiovascular hospitalization (HR 0.89, 95%CI 0.82-0.96; *p* = 0.003). Although statins seem to be promising in HFpEF, evidences are preliminary and should be confirmed in randomized trials.

### Are statins safe in patients with HF?

Major trials evaluating statins in HF demonstrate similar safety profiles in treatment or in placebo groups, even in the more severe patients, reassuring that statins are safe in this population ( 56 ). Since HF patients have a complex therapeutic regimen and polypharmacy, clinicians should be especially attentive to possible drug-to-drug interactions.

### Is the type of statin important in HF?

Differences between hydrophilic and lipophilic statins may be important in choosing a statin in patients with HF. While hydrophilic statins (pravastatin and rosuvastatin) are intrinsically hepato-selective, lipophilic statins (atorvastatin, simvastatin, fluvastatin, pitavastatin, and lovastatin) tend to be less selective to the liver and have higher exposure in extra-hepatic tissues ( 64 ).

A meta-analysis of 13 RCTs ( 64 ) indirectly compared a lipophilic statin (mostly atorvastatin) with a hydrophilic statin (rosuvastatin) with respect to outcomes in patients with HF. Atorvastatin was superior over rosuvastatin in cardiovascular mortality [OR 0.62 (95% CI 0.25, 0.99); *p* = 0.04], worsening of HF leading to hospitalization (OR 0.48, 95% CI 0.13-0.83, *p* = 0.00005), and all-cause mortality (OR 0.50, 95% CI 0.13-0.87, *p* = 0.0003). Statins seem to have no or little advantage in HFrEF, but may improve some clinical outcomes, including all-cause mortality, in patients with HFpEF. Clinical trials specifically in patients with HF and preserved EF are needed.

## Statin and liver disease

### Can statins cause liver disease?

Hepatoxicity of statins are rare and unpredictable, and the incidence is dose-related ( 65 ). In the Drug Reactions Advisory Committee between 1988 and 2010, 73 cases of drug-induced liver injury were associated with statins, corresponding to 1.2 episodes/100.000 users ( 66 ). Statins may elevate serum aminotransferase levels that are usually asymptomatic, occurring within the first year of treatment, and this elevation often resolves spontaneously. Persistent elevation of alanine aminotransferase levels of more than 3 times the upper limit of normality occurs with different statins in less than 3% of the cases ( 67 ).

All statins are cleared by the liver, and the rate of excretion depends on the lipophilicity of each statin. Statins with the highest (simvastatin and lovastatin) or modest (atorvastatin and fluvastatin) lipophilicity have a greater hepatic excretion rate, while the more hydrophilic ones (pravastatin and rosuvastatin) are excreted by the kidneys ( 68 ). A meta-analysis ( 68 ) revealed that RR for increased transaminases with high-intensity hydrophilic statins was 3.54 fold (95% CI 1.83-6.85) compared with low-intensity hydrophilic statins. On the other hand, when higher intensity lipophilic statins were compared with low-intensity statins, there was no association with the elevation of transaminases. The Food and Drug Administration revised statin safety and considered them safe for the liver. Monitoring transaminases during treatment is not recommended ( 69 ).

### Do statins reduce cardiovascular events in patients with non‐alcoholic fatty liver disease (NAFLD) and Non-Alcoholic Steato-Hepatitis (NASH)?

Patients with NAFLD/NASH usually have atherogenic dyslipidemia that may increase cardiovascular risk. A Swedish observational study ( 70 ) revealed an increased mortality in a 28-year follow-up period. Mortality in NASH/NAFLD cases proven by biopsy had increased by 69% compared with that in the general population, and the main cause of death (30%) was cardiovascular disease ( 70 ).

A post-hoc analysis in the GREACE study ( 71 ) evaluated the effect of atorvastatin in cardiovascular outcomes in patients with NAFLD. It was a prospective, randomized, open-label, survival study to compare the effect of statins on cardiovascular outcomes of patients with CAD and altered liver enzymes, with LDLc > 100 mg/dL and triglycerides < 400 mg/dL. The study enrolled 1,248 patients, aged < 75 years, with established coronary heart disease. Of these, 880 received for a statin and 720 did not for a mean of three years. After the follow-up period, there were 112 cardiovascular events, 13% in the statin group and 25% in the non-statin group, with a relative risk reduction of 49%, *p* < 0.0001.

### Can statins improve NAFLD?

The data regarding the favorable effect of statins on NAFLD improvement are still limited. A Cochrane systematic review ( 72 ) has shown that liver enzymes and image findings may improve with statins, but improvements in histology of NASH are still inconclusive. A prospective, 1-year, non-controlled pilot study evaluated rosuvastatin (10 mg) and lifestyle in patients with NASH. NASH resolution was evident in 19 of 20 biopsy-proven patients with metabolic syndrome and dyslipidemia, who received rosuvastatin ( 73 ). Liver enzymes normalized and ultrasonography showed absence of steatosis at the end of the study. Moreover, in the sub-analysis of GREACE study ( 71 ), there were 227 patients with raised concentrations of alanine transaminase (ALT) due to NASH, who received statins. At the end of the study, the group receiving statin displayed a significant reduction in ALT by 35% ( *p* < 0.001), while, the group not receiving statins displayed elevated ALT by 12% ( *p* < 0.01).

### Is there any benefit in using statins in chronc liver disease?

Recent observation indicates that statins may reduce portal hypertension. This has re-opened the debate on the possibility of considering the use of statins in chronic liver diseases. Simvastatin may improve portal hemodynamics by decreasing hepatic venous pressure gradient and ameliorating liver perfusion in patients with cirrhosis ( 74 ). Statins will confer protection against the development of cirrhosis and complications, such as portal hypertension, hepatic decompensation and hepato-cellular carcinoma ( 65 ). Simvastatin improves liver generation of nitric oxide and hepatic endothelial dysfunction in cirrhotic rats ( 74 ), decreases hepatic vein portal gradient (HVPG), and improves liver perfusion in patients with cirrhosis ( 75 ). In a trial including 59 patients with cirrhosis and portal hypertension, the patients were randomized for simvastatin 20-40 mg/day or placebo for one month, according to whether the patient was being treated with beta-adrenergic blockers or not. Simvastatin significantly reduced HVPG in comparison to placebo, independent of the concomitant use of beta-blockers, and it did not change systemic blood pressure. Only one patient in each group presented with raised hepatic enzymes.

Moreover, these data were reinforced by the findings that simvastatin may reduce mortality in chronic liver disease. A multicenter, randomized, double-blind, parallel trial ( 75 ) was conducted on 158 patients with Child-Pugh classes A or B cirrhosis on standard prophylaxis to prevent re-bleeding. This study assessed whether adding simvastatin to standard therapy could reduce re-bleeding and death after variceal bleeding in patients with cirrhosis. Seventeen (22%) patients in the placebo group died compared with 6 (9%) patients in the simvastatin group. Treatment with simvastatin was associated with a 61% reduction in the relative risk of death as compared with placebo ( 75 ) (HR 0.39, 95% CI: 0.15-0.99, *p* < 0.030). The addition of simvastatin to standard therapy was associated with a survival benefit in 24 months, although re-bleeding was similar between groups. These data suggest that simvastatin is potentially useful in prevention of complications in cirrhosis.

However, statin use in chronic liver disease should be considered with extreme care ( 76 ). Statins were studied in a trial including patients with decompensated cirrhosis. The LIVERHOPE-SAFETY study ( 77 ) was a randomized clinical trial in patients with Child-Pugh class B or C. The patients were randomly assigned to simvastatin 40 mg with rifaximin 1,200 mg, simvastatin 20 mg with rifaximin 1200 mg, or placebo. Treatment with simvastatin 40 mg in patients with decompensated cirrhosis was associated with a significant increase in adverse events requiring treatment withdrawal, particularly rhabdomyolysis, compared with simvastatin 20 mg ( 77 ). Chronic liver disease has long been considered a contra-indication for statin use. Although it does not necessarily preclude statin therapy, decompensated cirrhosis and acute liver failure are still formal contra-indications ( 76 ).

## Statins in the Elderly

### Do statins reduce mortality in elderly people?

The efficacy of statins in elderly people has been poorly addressed in randomized studies, but indirect data indicates that they may benefit from statins. Most evidence come from secondary outcomes and from sub-analysis of trials. A meta-analysis including 28 randomized trials ( 78 ) with 134,537 patients, divided people into six strata of age, ranging from 50 years to older than 75 years. Only 8% of the participants were above 75 years. Overall, there was 21% relative risk reduction in cardiovascular events per mmol/L of reduced LDLc (RR 0.79, 95% CI 0.77-0.81). Most importantly, there was little heterogeneity between the sub-groups with no trend for interaction with age, and the benefit occurred in all strata, independent of age. The main point is that, although there was statistical significance in the relative risk reduction, the absolute risk reduction was small. The number needed to treat was 98 (54-735) for elderly people with vascular disease and 132 for individuals without vascular disease with no mortality decrease.

However, the magnitude of the cardiovascular risk of the patient is of utmost importance for a real benefit. It is noteworthy that the higher the risk, the greater the chance of benefit. This occurs specially in patients with established cardiovascular disease. The most studied statin in higher risk patients is atorvastatin in high dose. Atorvastatin 80 mg was used in the PROVE IT TIMI 22 trial ( 79 ), the SAGE trial ( 80 ) and in the SPARCL trial ( 81 ), all with a considerable inclusion of elderly people. Briefly, the PROVE IT TIMI 22 compared atorvastatin 80 against pravastatin 40 mg in 4162 post-acute coronary syndrome patients. Among them, 634 individuals older than 70 years were included in a sub-analysis ( 82 ). The relative risk reduction in the elderly was high (around 40%), and the absolute risk reduction was 8%, with a NNT of 80 in two years.

The SAGE ( 80 ) trial also tested atorvastatin 80 mg against pravastatin 40 mg in elderly patients (65-85 years) with known myocardial ischemia. A total of 893 ambulatory coronary artery disease patients were included. The primary efficacy outcome (absolute change from baseline in total duration of ischemia at month 12) was significantly reduced in both atorvastatin and pravastatin groups at month 3 and month 12 ( *p* < 0.001 for each treatment group) with no significant difference between the groups. Interestingly the secondary outcome (all-cause mortality was reduced in 77% of the patients (HR 0.33, 95% CI 0.13-0.83, *p* = 0.014.

The original SPARCL study ( 80 ) randomized 4,731 patients with LDLc 100-150 mg/dL, with previous stroke, or transient ischemic attack (TIA), to receive atorvastatin 80 mg or placebo. A post-hoc sub-analysis ( 83 ) that divided patients into younger or older than 65 years, there were 1,153 individuals older than 65 years receiving atorvastatin 80 mg. The primary outcome (fatal or non-fatal stroke) was not met. However, there were many important risk reductions in the secondary outcomes list: stroke and TIA (HR 0.79, 95% IC 0.66-0.95, *p* = 0.01); coronary heart disease events (HR 0.61, 95% CI 0.45-0.81, p = 0.006) and revascularization (HR 0.55; 95% CI 0.40-0.77, *p* < 0.0005) ( 83 ). The magnitude of the benefit in the elderly depends largely on the reduction of LDLc, similar to that in younger people. However, in the elderly, the benefit may be attenuated due to the increase presence of important comorbities, such as HF, which are frequently present and do not benefit from statins.

### Are statins safe in the elderly?

Elderly people are prone to adverse events. The decrease in physiologic reserve, progressive frailty, polypharmacy, and the presence of severe comorbidities contribute to increasing adverse events associated to medications ( 84 ). Especially concerning polypharmacy, drug pharmacokinetics depends on body composition, albumin concentration, liver metabolism, and drug elimination, all of which change with age. This may cause an increase in drug concentration, elevating the risks of side-effect, especially for statin-associated muscle symptoms, the most commonly reported adverse effects in clinical practice, occurring in approximately 7%-29% of statin users ( 85 ).

When considering clinical trials, it has been demonstrated that statins may be similarly tolerated in older and younger adults. A large meta-analysis of randomized controlled trials ( 86 ) systematically evaluated the safety and tolerability of statin therapy in primary prevention in adults above 65 years of age. They identified 11 trials, including 18,192 participants, above 65 years of age (mean 73.7 years, 43% females). Compared with the placebo, statins did not increase the risks of muscle-related symptoms, total adverse events, or serious adverse effects. Reported adverse muscle symptoms in statin and placebo groups were 7.7% and 7.5%, respectively. Moreover, statins did not increase the risks of muscle-related symptoms (RR 1.01; 95% CI 0.90-1.12) and was not associated with permanent treatment discontinuations. No evidence of heterogeneity was observed in any of these outcomes, and there was no excess incidence of treatment discontinuations of statins relative to placebo in this population ( 86 ).

Although adverse effects can occur, statins are considered safe in the elderly, the adverse effects usually being mild and rarely dangerous ( 87 ). The prescription should be primarily guided by the cardiovascular risk (as more potent statins have additional benefits), then the limitations in their use due to intolerance mainly related to drug-drug interaction (DDI) should be given careful consideration. Clinicians, however, should consider avoiding statins in older frail adults unlikely to benefit due to frailty or a limited life expectancy.

### How to choose a statin in the elderly with polypharmacy?

Differences in statin metabolism have clinical significance and may determine DDIs. Isoenzyme CYP 3A4 of cytochrome P450 is the main catalyst for the metabolism of many drugs, including majority of the statins. The most frequent drug interactions are due to cometabolism by this enzyme ( 88 ). Lower risk of DDIs is seen with pravastatin because its metabolism is independent of P450, and with fluvastatin, which is metabolized via CYP2D9 ( 88 ). DDIs occurs when pharmacokinetics and/or pharmacodynamics of one medication is changed by concomitant administration of another drug, leading to a different effect from that expected of each medication when administrated alone. DDIs may cause either toxicity or reduced efficacy for one or both interacting drugs. They frequently occur due to induction or inhibition of metabolizing enzymes and/or transporters, resulting in changes in absorption, distribution, metabolism, or excretion. They can occur also by additive, synergistic, or antagonistic pharmacological effect ( 83 ) ( Table 4 ).

**Table 4:** Inhibitors and inducers of enzymatic pathways of statins metabolism

Pathway	Statin	Inhibitor	Inducer
CYP2C8	Fluvastatin; pitavastatin	Fluvoxamine, gemfibrozil, Ketoconazole, trimethoprim	Rifampicin
cYP2C9	Fluvastatin; pitavastatin; rosuvastatin	Amiodarone, cotrimoxazole, fluvoxamine, ketoconazole, metronidazole, oxandrolone, Voriconazole	Carbamazepine, rifampicin, Fenobarbital, phenytoin,
cYP3A4	Atorvastatin; lovastatin; Simvastatin	Alprazolam, amiodarone, amlodipine, azithromycin, cilostazol, cimetidine, ciprofloxacin, corticosteroids, clarithromycin, fluoxetine, fluconazole, tamoxifen, warfarin, ranitidine, tacrolimus, cyclosporine, danazol, tricyclic antidepressants	Barbiturates, carbamazepine, cyclophosphamide, dexa-methasone, omeprazole, phenobarbital, phenytoin, piogli-tazone, prednisone, rifampicin
mDRP	Atorvastatin; lovastatin; Pitavastatin; pravastatin; simvastatin	Cyclosporine, itraconazole, erythromycin, ketoconazole, verapamil, ritonavir	Rifampicin
oATP1B1	All statins	Clarithromycin, cyclosporine, Gemfibrozil, rifampicin, ritonavir	
uGT	Atorvastatin; lovastatin; pravastatin; simvastatin	Cyclosporine, gemfibrozil	Rifampicin

Inductors or inhibitors of CYP450 isoenzymes are a relevant cause of DDIs. Competitive inhibition between medications at the enzymatic level may increase plasma levels and lead to higher risk of adverse events, for example, hepatotoxicity and muscle complaints ( 89 ). On the other hand, CYP450 inducers may reduce statin plasma levels ( 90 ).

Clinicians should make a careful evaluation of possible DDIs when prescribing statins to patients receiving multiple drugs, consider different pharmacokinetic profiles among the statins to individualize treatment, weight the potential clinical benefits versus risks of adding a statin therapy, and select the best drug for that individual patient. Table 5 summarizes the potential interference of most used medications in the elderly with different types of statins.

**Table 5:** Statin recommendations for use with specific drugs

	Diltiazem, Verapamil Amlodipine	Amiodarone	Clopidogrel Prasugrel Warfarin Apixaban Edoxaban Rivaroxaban	Ticagrelor	Dabigatran	Gemfbrozil	Fenofibrate, Bezafibrate, Ciprofibrate
Atorvastatin						20 mg	10-20 mg
Lovastatin	20-40 mg	40 mg		40 mg			
Pitavastatin						2 mg	2 mg
Pravastatin							
Rosuvastatin						10 mg	10 mg
Simvastatin	10-20 mg	20 mg		40 mg			
Fluvastatin							

In conclusion, all statins lower LDLc and may be efficacious depending on their potencies and the cardiovascular risk of the patient. However, choosing statins wisely may minimize side-effects and improve compliance to treatment by the patient. When prescribing statins, one should first consider the potential cardiovascular risk benefit, but they can also adjust for the clinical conditions based on safety. Table 6 , summarizes the suggestions for each clinical situation thoroughly considered in this review.

**Table 6:** Statin recommendations in specific clinical situations

Clinical Condition	Suggested Statin	
	Primary Prevention	Secondary Prevention
Increased risk of new Diabetes	Pitavastatin	High potency
HIV using ART	Pitavastatin	Atorvastatin or rosuvastatin up to 20 mg a
Chronic Kidney Disease	Atorvastatin	Atorvastatin
Dialysis	Do not start statin b	Do not start statin b
Kidney transplant recipient:	Fluvastatin	High potency c
Heart transplant recipient	Pravastatin	High potency c
Heart Failure with preserved EF	Atorvastatin d	Atorvastatin d
NASH/NAFLD	Inconclusive	High potency
Decompensated Cirrhosis	Avoid statin	Avoid statin
Elderly	Pravastatin	High potency

a Avoid if using atazanavir with cobcistat.

b Keep statin, if already in use.

c Avoid if using with cyclosporine

d Data are preliminary. EF: ejection fraction; NASH: non-alcoholic steatohepatitis; NAFLD: non‐alcoholic fatty liver disease.
